# Supplementary material for: Anti-COVID-19 multi-epitope vaccine designs employing global viral genome sequences
Source: PeerJ. 2020 Aug 3;8:e9541. doi: 10.7717/peerj.9541 (PMC7409810; doi:10.7717/peerj.9541)
Supplement: Supplemental Information 3 [file peerj-08-9541-s003.docx]

| **Molecule** | **Active residues in HADDOCK** | **Passive residues in HADDOCK** |
| --- | --- | --- |
| Cov-I-vac | 4, 6, 7, 8, 10, 11, 12, 13, 14, 15, 16, 17, 19, 20, 21, 22, 23, 24, 25, 26, 27, 28, 29, 30, 31, 32, 33, 34, 35, 36, 37, 38, 39, 40, 41, 42, 43, 44, 45, 46, 47, 48, 49, 50, 51, 52, 55, 57, 58, 61, 65, 66, 67, 68, 69, 70, 82, 83, 84, 99, 107, 111, 112, 113, 114, 115, 128, 135, 138, 139, 140, 157, 165, 166, 167, 168, 191, 248, 281, 282, 283, 284, 285, 287, 288, 289, 291, 296, 301, 306, 307, 308, 309, 310, 311, 312, 313, 314, 315, 316, 317 | 1, 2, 3, 5, 9, 18, 53, 54, 59, 63, 64, 71, 72, 73, 75, 76, 77, 80, 81, 85, 86, 87, 95, 96, 97, 98, 100, 101, 102, 103, 104, 105, 106, 108, 109, 110, 118, 120, 121, 126, 129, 130, 131, 132, 133, 134, 136, 137, 141, 142, 156, 158, 159, 160, 161, 162, 163, 164, 169, 170, 172, 183, 185, 186, 189, 190, 192, 193, 195, 196, 211, 227, 245, 246, 247, 249, 250, 260, 261, 266, 267, 268, 269, 279, 280, 292, 293, 294, 295, 297, 298, 299, 300, 302, 303, 304, 305 |
| Cov-I-vac | 1, 2, 3, 5, 6, 9, 10, 16, 17, 18, 19, 20, 21, 22, 23, 24, 26, 33, 34, 35, 54, 55, 56, 57, 58, 59, 60, 61, 62, 63, 64, 65, 66, 68, 69, 70, 71, 72, 73, 74, 75, 76, 77, 78, 79, 80, 81, 82, 83, 84, 85, 86, 87, 88, 89, 90, 91, 92, 93, 94, 95, 96, 97, 98, 99, 100, 101, 102, 103, 105, 107, 108, 109, 110, 111, 113, 114, 116, 117, 143, 157, 170, 184, 185, 186, 193, 194, 195, 196, 199, 203, 204, 205, 212, 213, 215, 216, 219, 220, 221, 222, 223, 224, 230, 232, 253, 256, 257, 296 | 4, 7, 8, 11, 12, 13, 14, 15, 25, 27, 28, 29, 30, 31, 32, 36, 38, 39, 40, 41, 42, 46, 49, 50, 51, 52, 53, 67, 115, 118, 119, 120, 121, 122, 123, 125, 126, 140, 141, 142, 144, 145, 146, 154, 155, 156, 158, 159, 166, 168, 169, 171, 172, 173, 174, 175, 176, 177, 181, 182, 183, 187, 188, 189, 191, 192, 198, 200, 201, 202, 207, 208, 209, 210, 211, 214, 217, 218, 225, 226, 227, 228, 229, 233, 234, 235, 236, 237, 238, 241, 242, 248, 249, 250, 251, 252, 254, 255, 258, 259, 292, 293, 294, 295, 297, 298 |
| TLR-8 | 259, 261, 262, 263, 264, 265, 320, 322, 324, 346, 348, 349, 350, 351, 352, 353, 354, 375, 378, 403, 405, 427, 428, 429, 431, 432, 467, 468, 491, 492, 494, 495, 518, 520, 543, 653, 677, 678, 680, 701, 702, 723, 725, 726, 747 | 183, 231, 260, 266, 267, 268, 269, 273, 274, 293, 300, 301, 325, 328, 329, 355, 356, 358, 373, 380, 381, 407, 408, 409, 433, 434, 460, 461, 462, 465, 466, 469, 470, 474, 490, 497, 498, 513, 514, 516, 522, 523, 540, 541, 545, 546, 566, 567, 573, 574, 621, 626, 650, 652, 654, 655, 656, 675, 679, 682, 698, 699, 703, 705, 721, 722, 727, 729, 745, 749, 750, 771, 773 |
